# Benchmarking complete-to-partial point cloud registration techniques for laparoscopic surgery

**DOI:** 10.3389/frobt.2025.1702360

**Published:** 2025-11-17

**Authors:** Alberto Neri, Veronica Penza, Nazim Haouchine, Leonardo S. Mattos

**Affiliations:** 1 Biomedical Robotics Lab, Advanced Robotics, Istituto Italiano di Tecnologia, Genoa, Italy; 2 Department of Computer Science, Bioengineering, Robotics and Systems Engineering (DIBRIS), University of Genoa, Genova, Italy; 3 Harvard Medical School and Brigham and Women’s Hospital, Boston, MA, United States

**Keywords:** point cloud registration, deep learning, correspondences, computer-assisted surgery, laparoscopy

## Abstract

**Objective:**

Registering a preoperative 3D model of an organ with its actual anatomy viewed from an intraoperative video is a fundamental challenge in computer-assisted surgery, especially for surgical augmented reality. To address this, we present a benchmark of state-of-the-art deep learning point-cloud registration methods, offering a transparent evaluation of their generalizability to surgical scenarios and establishing a robust guideline for developing advanced non-rigid algorithms.

**Methods:**

We systematically evaluate traditional and deep learning GMM-based, correspondence-based, correspondence-free, matching-based, and liver-specific point cloud registration approaches on two surgical datasets: a deformed IRCAD liver set and DePoll dataset. We also propose our complete-to-partial point cloud registration framework that leverages keypoint extraction, overlap estimation, and a Transformer-based architecture, culminating in competitive registration results.

**Results:**

Experimental evaluations on deformed IRCAD tests reveal that most deep learning methods achieve good registration performances with TRE<10 mm, MAE(R) < 4 and MAE(t)<5 mm. On DePoll, however, performance drops dramatically due to the large deformations.

**Conclusion:**

In conclusion, deep-learning rigid registration methods remain reliable under small deformations and varying partiality but lose accuracy when faced with severe non-rigid changes. To overcome this, future work should focus on building non-rigid registration architectures that preserve the strengths of self-, cross-attention and overlap modules while enhancing correspondence estimation to handle large deformations in laparoscopic surgery.

## 1 Introduction

Augmented Reality (AR) integrates computer-generated images with the real world to enhance the user’s perception. In surgery, AR systems overlay patient-specific 3D models (for example, organs, tumours, and vessels) directly onto the operative view, giving surgeons persistent, intuitive access to preoperative imaging information. This can aid intraoperative tasks such as tumour localisation, margin assessment, and avoidance of critical vasculature, with potential benefits including shorter operative times and fewer complications when AR is used effectively (Prasad et al., 2024). In recent years, AR has been progressively adopted in various surgical settings, including neurosurgery, orthopaedics, and laparoscopy (Bernhardt et al., 2017). However, challenges arise from the dynamic nature of organ tissues, in particular in abdominal surgery, patient positioning, pneumoperitoneum insufflation, and physiological motion all cause global shifts, while direct instrument–tissue interactions produce highly localized and often large deformations (Bernhardt et al., 2017). These factors can cause the intraoperative images to differ from the preoperative images, which capture the anatomy prior to surgery. Aligning preoperative models with intraoperative images during laparoscopy remains a key focus of research, with many challenges still unresolved (Neri et al., 2025a).

**FIGURE 1:**
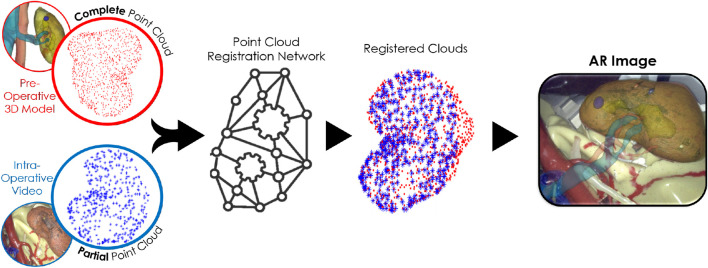
DL-based point cloud registration methods use organ point clouds extracted from the preoperative model and intraoperative stereo-video as inputs and estimate the transformation that aligns the two point clouds, allowing the creation of the AR image for surgical navigation.

Conventional surface-based methods align the preoperative model (surface mesh or point cloud) with intraoperative data using geometric shape information. This process relies on computer vision algorithms to reconstruct the intraoperative 3D surface and typically employs techniques like Iterative Closest Point (ICP) (Besl and McKay, 1992), along with tracking and matching algorithms (Puerto-Souza et al., 2014), to perform the registration. However, it faces challenges such as incomplete reconstructions due to occlusions and lack of distinctive features, further worsened by complex, texture-less, and deformable scenes (Marques et al., 2015). To address the limitations of traditional surface-based methods, various algorithms are being developed incorporating Deep Learning (DL). One approach involves hybrid DL methods, which enhance conventional surface-based techniques by integrating DL at specific stages, thereby improving registration effectiveness and outcomes. For instance, DL can be applied to tasks such as image segmentation (Zhang et al., 2022), intraoperative surface reconstruction (Luo et al., 2020), or feature extraction (Labrunie et al., 2022). Alternatively, fully DL-based algorithms, such as end-to-end networks for point cloud registration (Huang et al., 2021), have been employed. These networks take two point clouds as inputs and generate the transformation required to align them (Figure 1).

Recently, a limited number of studies have been published in this last category. They can mainly be divided into two classes: correspondence-free, which do not require the prediction of one-to-one point correspondences, and correspondence-based, which explicitly predict such correspondences.

A correspondence-free approach is (Guan et al., 2023), which introduced the first deep learning-based approach for 3D-3D laparoscopic liver registration. The method builds on OMNet (Xu et al., 2021) and does not utilise Transformers, instead leveraging local and global feature extraction to learn overlapping masks from the preoperative 3D model and the intraoperative reconstruction. These masks are used to filter out non-overlapping regions and standardise the point clouds before aligning the overlapping areas. Thus, this method directly predicts the final transformation without estimating the point correspondences.

LiverMatch (Yang et al., 2023) is a correspondence-based method to register complete-to-partial synthetic point clouds of liver anatomy. The method consists of a transformer encoder-decoder network that learns feature descriptors, which are then fed to a matching module that predicts point correspondences. The promising results suggest that estimating correspondences between sets of point cloud descriptors leads to good registration results, even in the presence of small deformations.

Dai et al. (2025) introduce a correspondence-based method that uses a Transformer encoder–decoder architecture. Unlike LiverMatch, which applies the Transformer to encoder features, they employ a geometric Transformer (Qin et al., 2023) on decoded features and complement it with deep graph matching guided by overlap masks to refine correspondence quality.

Finally, Zhang et al. (2024) propose KCR-Net, a correspondence-based method built on an encoder-only Transformer. KCR-Net first extracts keypoint descriptors using a Neighbourhood Feature Fusion Module (NFFM) that employs both self- and cross-attention, and then estimates keypoint correspondences. Unlike (Yang et al., 2023) and (Dai et al., 2025), which recover the final transform from dense correspondences, KCR-Net computes the transformation just from the sparse keypoint matches.

Among these, LiverMatch is the only open-source algorithm.

These approaches are applicable to surgical guidance, since point clouds can be extracted from both preoperative images (e.g., CT scans) and intraoperative images (e.g., stereo cameras), as shown in Figure 1. However, although these point clouds represent the same organ geometry, they differ not only due to deformations, but also because of varying levels of partiality and noise. For instance, the registration to be solved is complete-to-partial; in fact, point clouds derived from CTs (obtained from well-established segmentation techniques (Isensee et al., 2021)) are *complete*, noise-free and dense. In contrast, stereo-camera point clouds are *partial*, capturing only the surface regions visible to the camera ( ≈ 30% of the organ (Koo et al., 2022)), and typically noisy, even with the latest 3D reconstruction methods (Zha et al., 2023).

As presented in (Neri et al., 2025a), various end-to-end rigid registration approaches exist, but current evaluations remain restricted mainly to partial-to-partial experiments on classical vision datasets such as ModelNet40 (Wu et al., 2015), which contains rigid, noise-free objects; consequently, little is known about how these methods behave under the conditions that characterise surgical point clouds. To address this gap, we provide the first systematic benchmarking of state-of-the-art registration networks in a surgical scenario, assessing their robustness and limitations in synthetic intraoperative settings, including complete-to-partial matches, noise and soft-tissue deformation. Among the methods compared, we introduce a refined correspondence-based registration method with an improved overlap-estimation module that yields more accurate correspondences and competitive performance against current baselines. Although all tested algorithms perform rigid registration while the underlying problem is non-rigid, rigid alignment is a useful intermediate step: (i) it brings the two clouds into closer correspondence for a subsequent non-rigid refinement, and (ii) rigid architectures provide a convenient backbone that can be extended to predict dense deformations. Because the solution quality depends strongly on the magnitude of deformation, our evaluation progresses from small deformations across varying levels of partiality to the extreme cases represented in the DePoll dataset (Modrzejewski et al., 2019) (large deformations). Overall, the benchmark isolates the essential building blocks for reliable registration and provides a practical guideline for developing new methods in surgical scenarios.

## 2 Benchmarking protocol

### 2.1 Problem formulation

Let $X\in R^{M\times 3}$ be the complete point cloud of the organ of interest (from preoperative planning), and let $Y\in R^{N\times 3}$ be a partial point cloud of the same organ (e.g., captured using an endoscopic camera), where N≪M . We define $X_{\text{visible}}\subset X$ as the subset of points in X that correspond to the partial cloud Y , so that $X_{\text{visible}}\approx Y$ . The goal of point cloud registration is to determine the unknown rigid transformation, composed of a rotation R∈SO(3) and a translation $t\in R^{3}$ , that aligns X with Y , i.e., we seek a transformation T such that $T\left(X_{\text{visible}}\right)=\left(RX_{\text{visible}}+t\right)\approx Y$ .

### 2.2 Competing methods

The following sections present the state-of-the-art open-source methods we evaluated to identify the baseline that best generalizes to real surgical scenarios. To ensure fair comparisons we selected methods according to three criteria: (a) they operate on 3D point clouds (rather than multi-modal pipelines that require image-based tracking or fiducials); (b) their implementations are open-source and can be adapted to the complete-to-partial evaluation setting; and (c) they produce the same output object (a global rigid transformation) so that all methods can be assessed with the same metrics. Accordingly, the following sections first review traditional (non-deep learning) registration techniques and then cover deep learning approaches. Because publicly available, deep learning complete-to-partial registration methods are scarce (e.g., LiverMatch is an exception), we primarily selected partial-to-partial algorithms. This category is the closest available match to our complete-to-partial scenario and can be adapted to our benchmark under the constraints above. Table 1 summarizes all the selected methods.

**TABLE 1:** Summary of the methods compared.

Name	Registration type	Approach	Main components
*ICP*	Rigid	Iterative	Point-to-Point
*DCP*	Rigid	Iterative, Probabilistic	1 GMM
*GMMReg*	Rigid	Iterative, Probabilistic	2 GMM
*OGMM*	Rigid	GMM-Based	Transformers, Overlap Score, 2 GMM
*OMNet*	Rigid	Correspondence-Free	Overlap Masks, Global Features
*Lepard*	Matching	Correspondence-Based	Transformers, Repositioning
*LiverMatch*	Matching	Correspondence-Based	Transformers, Visibility Score
*Ours*	Rigid	Correspondence-Based	Transformers, Overlap Score

#### 2.2.1 Traditional Methods

We classify “Traditional Methods” as those that do not rely on deep learning. Among these, we have selected several popular approaches, including Iterative Closest Point (ICP) (Besl and McKay, 1992), Coherent Point Drift (CPD) (Myronenko and Song, 2010), and Gaussian Mixture Models Point Set Registration (GMMReg) (Jian and Vemuri, 2011).

##### 2.2.1.1 ICP

ICP is a widely used rigid registration algorithm that aligns two point clouds by iteratively minimizing the distance between corresponding points. In each iteration, the algorithm identifies the nearest neighbours between the datasets and computes the optimal transformation that reduces the alignment error.

##### 2.2.1.2 CPD

CPD is a probabilistic point cloud registration algorithm that treats one point set as centroids of a Gaussian mixture model while aligning it to the other point set. It enforces smooth motion by assuming nearby points to move coherently, which helps maintain local geometric structure during the transformation.

##### 2.2.1.3 GMMReg

GMMReg is a probabilistic framework representing both input point sets as Gaussian mixture models. In this formulation, the point set registration task is transformed into aligning the two mixtures to minimize a statistical discrepancy measure between them.

#### 2.2.2 Deep learning methods

According to the classification proposed in (Neri et al., 2025a), we selected deep learning point cloud registration methods belonging to different categories such as: correspondence-free, GMM-based, correspondence-based and liver-specific.

##### 2.2.2.1 Correspondence-free, OMNet

We selected OMNet (Xu et al., 2021) to represent correspondence-free deep learning methods. Its core concept involves using overlapping masks to discard non-overlapping points, thereby retaining only the overlapping regions that are most useful for estimating the transformation through global feature regression. Notably, OMNet inspired the work of Guan et al. (2023), which improved OMNet local feature extraction following the RPMNet (Yew and Lee, 2020) model. Despite that, Guan et al. (2023) closed-source nature led us to opt for testing OMNet instead.

##### 2.2.2.2 GMM-based, OGMM

OGMM (Mei et al., 2023) introduces an overlap-guided probabilistic registration approach that estimates the optimal transformation by matching Gaussian Mixture Model parameters. Similarly to GMMReg, the method reformulates registration by aligning two Gaussian mixtures to minimize statistical discrepancies. Additionally, a Transformer-based detection module is employed to identify overlapping regions, using the resulting overlap scores to guide the GMM representation and alignment of the input point clouds.

##### 2.2.2.3 Correspondence-based, lepard

Lepard (Li and Harada, 2022) is a learning-based method for partial point cloud matching in rigid and deformable scenes, predicting correspondences that are later registered using ICP or N-ICP. Its architecture combines a fully convolutional feature extractor (KPFCN) with a Transformer employing self- and cross-attention to compute a differentiable similarity matrix. A repositioning module further refines the relative positions between point clouds, enhancing cross-attention and matching effectiveness, which makes Lepard one of the leading methods in non-rigid point cloud registration.

##### 2.2.2.4 Liver-specific, liver match

LiverMatch (Yang et al., 2023) stands out as one of the few open-source deep-learning approaches designed explicitly for surgical laparoscopic registration. It employs an encoder-decoder architecture enriched with self- and cross-attention mechanisms to extract point features that are then used to compute a similarity matrix. This matrix, in combination with a visibility score, is utilized to predict correspondences between the two point clouds. Following correspondence determination, registration is carried out using ICP. To train the network to handle deformations, the authors generated a synthetic dataset by applying deformations and cropping techniques to 16 livers from the 3D-IRCADb-01 dataset (Soler et al., 2010).

##### 2.2.2.5 Refined RegTR (ours)

**FIGURE 2:**
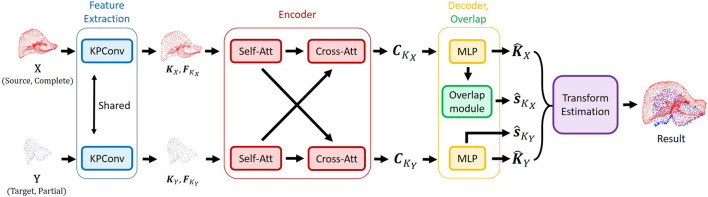
The network leverages KPConv to extract keypoints and their associated features. These features are further refined using self- and cross-attention mechanisms and then passed to the decoder, which predicts overlap scores and the corresponding keypoints. This information is ultimately used to estimate the final transformation.

We extend RegTR (Yew and Lee, 2022) with a refined overlap-estimation module designed to enhance the accuracy in predicting the final transformation (Figure 2). RegTR leverages keypoint features, which are fundamental since correspondences are determined among these keypoints rather than using all points. A KPConv backbone is employed to extract a reduced set of keypoints ( $K_{X}\in R^{M^{\prime }\times 3}$ , $K_{Y}\in R^{N^{\prime }\times 3}$ ) and their associated features ( $F_{K_{X}}\in R^{M^{\prime }\times D}$ , $F_{K_{Y}}\in R^{N^{\prime }\times D}$ ) from the input clouds, which are then projected to a lower dimension (256) and enriched with sinusoidal positional encoding. These components are fed into a transformer cross-encoder, using both self-attention (within each cloud) and cross-attention (across clouds), to produce conditioned features ( $C_{K_{X}}\in R^{M^{\prime }\times d}$ and $\;C_{K_{Y}}\in R^{N^{\prime }\times d}$ ) that effectively identify accurate correspondences and filter outliers, serving a role similar to RANSAC in traditional approaches.

The conditioned features are fed into a two-layer MLP to predict the transformed keypoint coordinates ( ${\hat{K}}_{X}\in R^{M^{\prime }\times 3}$ and ${\hat{K}}_{Y}\in R^{M^{\prime }\times 3}$ ).

**Overlap Module**. In parallel with the decoder, overlap scores for the two point clouds are predicted. The overlap score, denoted as $\hat{s}=\left[{\hat{s}}_{K_{X}},{\hat{s}}_{K_{Y}}\right]$ , represents the probability that a keypoint belongs to the overlap region. Unlike (Yew and Lee, 2022), where overlap scores are predicted for both point clouds, our method predicts the overlap score only for the complete point cloud X . Indeed, since the problem we are solving is a complete-to-partial registration, we know by definition that the partial point cloud is fully contained within the complete one ( $X_{\text{visible}}\subset X$ and $Y\approx X_{\text{visible}}$ ). Consequently, every point in Y belongs to the overlap area, and its overlap score is 1. Therefore, predicting overlap scores for the partial cloud provides no additional information but does introduce an extra source of estimator error: imperfect predictions on Y can produce false negatives or false positives that harm the overall performance.

To predict which points of the complete model are observed in the partial scan, we apply a linear fully-connected layer with an elementwise sigmoid activation to produce per-point overlap scores as follows (Equation 1):

$$\hat{s}=\left\{\begin{array}{c} {\hat{s}}_{K_{X}}=1/\left(1+e^{-\left(C_{K_{X}}w_{1}+b_{1}\right)}\right)\\ {\hat{s}}_{K_{Y}}=1_{N^{\prime }}={\left(1,1,\ldots ,1\right)}^{⊤}\in R^{N^{\prime }\times 1} \end{array}\right.$$ (1)

where $w_{1}$ and $b_{1}$ are learnable weights and biases parameters and $C_{K_{X}}$ are the conditioned features. ${\hat{s}}_{K_{Y}}=1_{N^{\prime }}$ ensures that all points in the partial cloud are part of the overlap region (i.e., overlap score =1 ).

**Transformation prediction**. Finally, the predicted transformed keypoint coordinates are concatenated to form correspondence pairs. Unlike methods such as Lepard and LiverMatch, which rely on similarity matrices and matching losses, RegTR directly predicts the transformed coordinates and treats them as correspondences for final transform estimation; therefore, it does not construct a similarity matrix or depend on correspondence supervision. The rigid transformation is estimated by leveraging the correspondences and overlap scores and minimizing the weighted sum of squared distances between the corresponding points. We solved it using a weighted variant (Gojcic et al., 2020) of the Kabsch-Umeyama algorithm (Umeyama, 1991).

**Losses and Optimization**. Our method employs a weighted sum of three losses similar to (Yew and Lee, 2022): (i) a registration loss that minimizes the error between the predicted transformed keypoint positions and their ground truth counterparts, weighted by the overlap confidence; (ii) a conditioned feature loss that encourages the network to consider geometric properties and to distinguish correct correspondences from incorrect ones in the context of feature matching; and (iii) an overlap loss designed to optimize the overlap scores, which measure the confidence that a keypoint from X has a valid correspondence in the overlapping region of Y . Early stopping is applied: training terminates if the validation loss does not improve for 12 consecutive epochs. On an NVIDIA Tesla V100 GPU, the full training run takes approximately 5 h.

### 2.3 Dataset and pre-processing

We employed two datasets with different deformation magnitudes to assess how rigid registration algorithms generalize to non-rigid scenarios. First, we generated a customized complete-to-partial version of the 3D-IRCADb-01 dataset, incorporating small random deformations, noise, and varying levels of partiality. To further stress-test the algorithms, we also used the DePoll dataset, which features large deformations, irregular noise, and severe partiality.

#### 2.3.1 Deformed IRCAD

**FIGURE 3:**
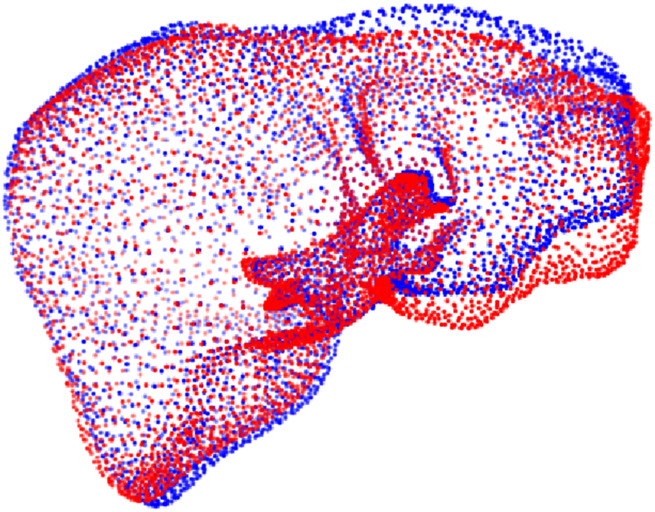
An example of synthetic deformation applied to the complete preoperative liver (red), where the right lobe is shifted upward (blue). The final intraoperative cloud is then cropped from the deformed model (blue).

The original IRCAD dataset (Soler et al., 2010) consists of 3D CT scans from 10 women and 10 men, with hepatic tumors present in 75% of the cases. The dataset also contains the VTK models of each liver. For each VTK, we extracted the point cloud that describes its surface and sampled 3,500 points, which provided a balance between geometric detail and GPU memory constraints. The points were normalized in the range [-1, 1] across all three axes, producing 20 different source point clouds. Subsequently, inspired by the approaches proposed by Livermatch (Yang et al., 2023) and (Dai et al., 2025), we generate several corresponding partial, deformed targets for each source point cloud. Differently from them, our pipeline employs the As-Rigid-As-Possible (ARAP) deformation algorithm from Open3D (Sorkine and Alexa, 2007), which lets us deform the liver mesh via user-defined control and anchor points (Neri et al., 2025b). Specifically, we randomly choose one of the two liver lobes and apply a random translation of up to ± 25 mm along both the x and z-axes. This range, corresponding to 2.5 cm in real scale, was selected empirically to simulate small yet realistic liver deformations (Figure 3); considering that an adult liver measures roughly 20 cm in width (Jones J and Walizai, 2009).

To simulate realistic endoscopic views, we place a virtual camera aimed at the organ’s visible surface. We sample random camera positions in spherical coordinates, constraining polar and azimuthal angles to match typical intraoperative ranges. For each point on the surface, we compute the dot product between the camera direction and the point normal, retaining only 15% of points whose angle to the camera is less than 80°(empirically found). Finally, we apply a random rigid transformation to the target, with a rotation up to 45°and a translation in the [-50 mm, 50 mm] range. We add element-wise Gaussian noise with zero mean and a standard deviation of 0.01 to each point in every dimension. Regarding seed management, a random seed is assigned to each training pair. This seed controls the generation of all stochastic parameters, including the selected lobe to deform, the magnitude of its displacement, the camera viewpoint, the applied rigid transformation, and the noise level.

For the training set, we used 17 livers (i.e., livers numbered 3 through 20) as source point clouds and generated 560 partial target clouds for each, yielding a total of 10,080 pairs. Using the same workflow, we created 4 testsets based on the remaining 2 livers (i.e., liver 1 and 2). Each set includes 50 partial targets per source (100 pairs total). The variation arises from the crop ratio: 5%, 10%, 15%, or 25%, chosen to mimic different levels of intraoperative organ exposure, and from the random deformations generated as in training. The selected levels of partiality reflect the surgical context, where the algorithm must handle varying exposure of the organ to provide AR guidance. In practice, surgeons typically expose only about 20%–30% of the organ surface during a procedure (Koo et al., 2022; Benincasa et al., 2008); we therefore included lower partiality levels ( < 20%) to stress-test the robustness of the evaluated algorithms under particularly challenging visibility conditions. Partiality below 20% poses challenges due to insufficient discriminative features in the intraoperative point cloud (Benincasa et al., 2008). Although surgeons generally expose as much of the organ as possible before resection, visibility rarely exceeds 50% of the surface area, as the opposite side remains occluded.

Because these datasets are synthetically generated, we retain complete ground-truth annotations, including the applied rigid transformations, known point correspondences, and overlap scores. Finally, to enable the computation of the Target Registration Error (TRE), we choose *n* landmark points on the original (source) mesh and identify their exact correspondences on the deformed (target) mesh. To ensure these points are not part of the input clouds provided to the registration algorithm, we select them from the cropped-out regions, applying farthest point sampling.

Code and data to reproduce our deformed IRCAD dataset are available at: https://github.com/Alberto-Neri/Laparoscopic_Organ_Deformation_wARAP.

#### 2.3.2 DePoll

**FIGURE 4:**
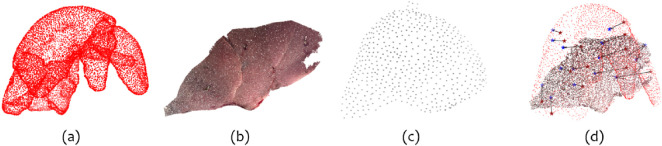
**(a)** The complete preoperative pig liver point cloud with segmented lobes. **(b)** The intraoperative video reconstructed point cloud (partial), relative to case 1. **(c)** The intraoperative CT scan point cloud (partial), relative to case 1. **(d)** Point cloud (a) and (b) registered with the ground truth rigid transformation. The dark red stars represent the surface marker on the preoperative anatomy; the blue stars represent the same surface markers on the intraoperative anatomy (after non-rigid deformations). The black arrows show the displacements between the corresponding markers, representing the effect of non-rigid deformation. The GT TRE is computed as the sum of the distances indicated by the black arrows. For simplicity, the figure shows only surface markers and the two clouds with a lower resolution.

DePoll (Deformable Porcine Laparoscopic Liver) dataset (Modrzejewski et al., 2019) comprises preoperative and intraoperative pig liver surface point cloud data under different deformation states. Specifically, it includes a preoperative point cloud of the pig liver, which is complete and derived from a CT scan. Regarding the intraoperative data, there are 13 cases of the same liver under various deformation conditions. Each case contains a partial point cloud extracted from an intraoperative CT scan and a partial point cloud obtained from video reconstruction. Figures 4a–c show one representative case, displaying the complete preoperative liver point cloud alongside two corresponding intraoperative partial reconstructions–one from CT and one from video. To normalize the points in the range [-1, 1], we applied min-max normalization using the maximum and minimum values of the complete preoperative point cloud. The dataset authors obtained the ground truth registration using pre- and intraoperative markers and the point clouds are provided pre-registered. Moreover, since the data come from two different sensors (i.e., CT scan and endoscope), they already exhibit noise and density variations. For this reason, the only pre-processing we applied was generating a random rigid transformation within the range proposed for IRCAD dataset.

### 2.4 Metrics

To evaluate the rigid registration quality, we compute the mean absolute error (MAE ( R ), MAE ( $\left.t\right)$ ) between the predicted and the ground truth values for both the rotation angle and translation. All methods directly estimate a rigid transformation, except Lepard and LiverMatch, which output point-to-point correspondences. For these two, we recover the rigid pose by feeding their correspondences into Open3D’s RANSAC-ICP routine, as proposed by LiverMatch. We set the max_correspondence_distance parameter to 0.05, producing the best alignment results.

We also report the TRE (mm) for each experiment. For the IRCAD dataset, we use the landmark coordinates identified during preprocessing (see Section 2.3.1), while for DePoll we rely on the preoperative and intraoperative markers provided by the dataset authors.

### 2.5 Experimental setup

ICP was run with an identity initialization, a convergence threshold of 0.001, and a maximum of 30 iterations. CPD was configured with the same threshold and up to 50 iterations. For GMMReg, the number of Gaussian components was set equal to the number of points in the target cloud.

All deep learning models were trained on our deformed IRCAD dataset (15% crop ratio), with minor code adjustments and hyperparameter tuning to ensure optimal convergence. For OMNet, which expects two partial point cloud of equal size, we padded the smaller target clouds to preserve its input structure and replaced all BatchNorm layers with GroupNorm to stabilize training with our small batch sizes. We also substituted the authors’ overlap score estimation with our ground-truth overlap scores, which are better suited to deformed data. Each of these adjustments led to significantly improved convergence. Similar modifications were applied to OGMM, yielding performance gains but still less optimal convergence; in this case, we opted not to introduce further changes to respect the original design. Finally, we improved Lepard and LiverMatch’s convergence by setting the hyperparameter first_subsampling_dl to 0.03.

All algorithms were evaluated on our four IRCAD deformed test sets, each defined by a different crop ratio (5%, 10%, 15%, and 25%), to mimic varying levels of intraoperative organ exposure. We also tested each algorithm on the DePoll dataset for further generalization evaluation on large deformations.

## 3 Results

### 3.1 Evaluation on the IRCAD deformed dataset

**FIGURE 5:**
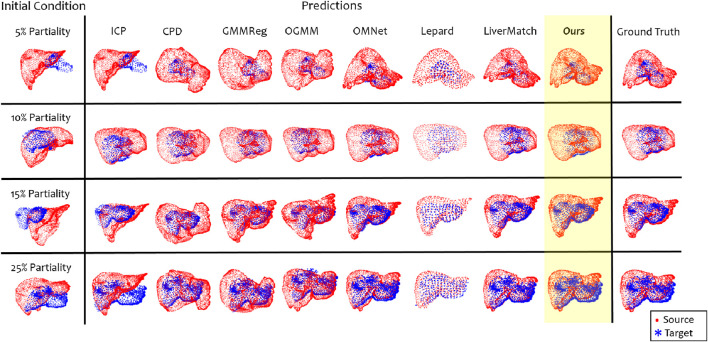
Each model’s qualitative results were evaluated under four distinct levels of partiality (i.e., 5%, 10%, 15% and 25%). The first column illustrates the initial conditions (i.e., after pre-processing), the following shows the predicted registration, and the last provides the ground truth registration.

Figure 5 presents the algorithms’ qualitative results at varying partiality levels.

Table 2 shows the performance (mean ± std) of the algorithms on the IRCAD deformed test set at the four partiality levels analyzed.

**TABLE 2:** Performance metrics at different levels of partiality.

OGMM	5% partiality			10% partiality			15% partiality			25% partiality		
Algorithm	TRE (mm)	MAE(R) (°)	MAE(t) (mm)	TRE (mm)	MAE(R) (°)	MAE(t) (mm)	TRE (mm)	MAE(R) (°)	MAE(t) (mm)	TRE (mm)	MAE(R) (°)	MAE(t) (mm)
ICP	62.69±27.79	22.60±8.21	21.34±7.11	62.19±28.44	24.03±8.76	19.95±7.37	66.15±29.51	25.30±8.30	22.56±6.94	65.22±28.61	25.61±6.85	21.69±6.42
CPD	80.05±30.55	43.09±21.93	19.43±6.68	79.52±32.70	46.62±24.12	20.82±5.64	78.44±34.78	47.09±24.52	22.56±6.07	66.33±39.27	38.15±27.39	20.82±7.72
GMMReg	67.51±29.66	21.21±9.38	27.50±7.03	66.36±35.55	21.95±11.29	26.89±8.68	65.62±36.07	21.28±13.05	26.03±8.07	55.72±33.22	19.62±13.09	21.69±7.98
	82.55±34.07	21.99±9.65	32.97±5.90	73.60±27.76	16.54±6.63	29.50±5.38	65.40±24.83	14.51±6.61	26.89±5.38	31.14±17.91	9.03±3.57	10.41±5.55
OMNet	23.49±14.14	9.00±4.33	7.20±3.64	15.23±8.36	5.77±2.58	5.29±2.17	11.72±6.45	4.37±1.95	3.82±1.91	10.45±4.81	3.86±1.76	3.73±1.82
Lepard	27.03±39.44	10.94±18.04	10.58±15.79	9.82 ± 8.59	3.91±2.30	4.86±3.04	7.45 ± 5.49	3.21±1.95	3.64 ± 2.08	7.41±6.69	3.27±1.68	3.04±2.26
LiverMatch	18.69±28.58	7.47 ± 6.09	8.41±10.67	7.91±5.90	3.64±2.03	4.68 ± 2.17	6.39±4.54	3.08 ± 1.91	3.73±2.00	4.81±3.21	2.78 ± 1.52	2.78±1.47
***Ours***	22.36 ± 12.04	6.60±4.14	7.72 ± 4.77	13.01±6.07	3.79 ± 2.25	4.25±2.00	8.49±2.62	2.85±1.32	2.60±1.13	6.78 ± 2.08	2.33±1.06	2.86 ± 0.87
*GT*	6.45±5.62	0.00±0.00	0.00±0.00	6.17±5.18	0.00±0.00	0.00±0.00	5.37±4.82	0.00±0.00	0.00±0.00	5.01±4.40	0.00±0.00	0.00±0.00

Bold represents best values, underline represents second best values.

The last table row describes the ground truth error, which for TRE represents the residual non-rigid component. In particular, the TRE remains nonzero because it is measured between preoperative markers and their intraoperative counterparts, which have undergone both a rigid transformation and non-rigid deformation. Since all evaluated algorithms perform solely rigid registration, they inherently cannot correct for the non-rigid component, resulting in a residual error consistent with this ground-truth baseline (same concept of Figure 4D representing DePoll dataset). While TRE may not fully capture performance under our scenario, it remains the primary metric in surgical applications. Therefore, we include TRE results for all rigid registration methods to transparently evaluate their performance and highlight their inherent limitations under these challenging conditions.

We first analyze the 15% partiality test set, which uses the same level of partiality as the training data but with different liver anatomies. In this scenario, our method achieves the third-lowest TRE ( 8.49mm±2.62mm ) yet remains highly competitive, as evidenced by its lower standard deviation and best performance across other rigid registration metrics (i.e.,: MAE(R)=2.58°±1.32° and MAE(t)=2.60mm±1.13mm ).

Across the remaining partiality levels, models must generalize not only to new liver anatomies but also to varying crop ratios. Our method remains highly robust, consistently ranking first or second, while conventional rigid techniques struggle with the complete-to-partial registration challenge. In particular, GMMReg at 25% partiality shows TRE=55.72mm±33.22mm , MAE(R)=19.62°±13.09° and MAE(t)=21.69mm±7.98mm . Even deep learning partial-to-partial approaches exhibit limited generalizability, with OGMM showing the greatest performance drop (at 25% partiality: TRE=31.14mm±17.91mm , MAE(R)=9.03°±3.57° and MAE(t)=10.41mm±5.55mm ). All methods degrade at low visibility (small target area), whereas higher partiality (e.g., 25%) yields noticeably better registration accuracy. For instance, at 25% partiality, our method achieves TRE=6.78mm±2.08mm , MAE(R)=2.33°±1.06° and MAE(t)=2.86mm±0.87mm .

Examining the qualitative results in Figure 5, we observe that some predictions closely match the ground truth; however, the models often fail to capture non-rigid deformations and instead bias the target’s contours to match the preoperative edges.

### 3.2 Evaluation on the DePoll dataset

To further assess generalization, we evaluated each model across all 13 cases in the DePoll dataset using the same weights trained on the deformed IRCAD data. Differently from previous studies that register the intraoperative CT–derived point cloud to the video-based reconstruction ((Guan et al., 2023; Zhang et al., 2024; Dai et al., 2025)), we perform complete-to-partial registration by treating the preoperative CT–derived liver model as the “complete” source and the video-based reconstruction as the “partial” target. The critical difference is the source cloud: the intraoperative CT scans deliver a partial and non-rigidly deformed liver model (Figure 4C), whereas the preoperative model (Figure 4A) describes the full organ before any deformation. Although these conditions are more demanding, we believe they more accurately reflect surgical practice, where a complete preoperative 3D model is registered to a partial, intraoperative surface reconstruction.

**FIGURE 6:**
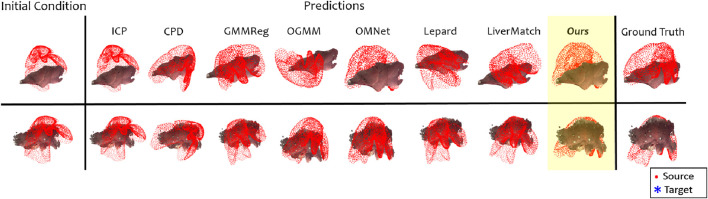
Qualitative registration results on the DePoll dataset, each row represents a different case. The inputs used were the preoperative complete point cloud and the intraoperative partial point cloud reconstructed from the video.

Figure 6 shows some qualitative registration results produced by each algorithm tested. Table 3 presents the quantitative results as the mean ± std on the 13 test cases.

**TABLE 3:** Average performance metrics on DePoll dataset.

Algorithm	TRE (mm)	MAE(R) (°)	MAE(t) (mm)
ICP	60.16±28.96	24.09±7.78	21.61±6.31
CPD	58.09±26.81	25.47±7.90	16.48±6.70
GMMReg	56.23±28.55	21.41±5.45	14.98±7.10
OGMM	68.57±38.05	28.61±22.55	27.20±12.54
OMNet	42.85 ± 25.65	16.05 ± 5.68	11.28±3.94
Lepard	72.47±32.06	24.09±7.78	22.08±8.44
LiverMatch	54.47±28.54	17.87±6.19	17.11±8.44
*Ours*	42.10±23.74	14.66±5.52	12.22 ± 5.52
*GT*	29.58±22.38	0.00±0.00	0.00±0.00

Bold represents best values, underline represents second best values.

Although our approach outperforms existing methods, its performance remains insufficient to overcome this challenge entirely. With a MAE(R) of 14.66°±5.52° and MAE(t) of 12.22mm±5.52mm , residual misalignments are frequently large enough to be visually perceptible. The first row of Figure 6 shows one of the best examples: both our method and OMNet approximate the ground truth, although a noticeable rotational offset remains. This case also highlights how intraoperative anatomy can differ substantially from the preoperative scan: achieving the target alignment here requires a large leftward displacement of all three liver lobes. In contrast, the second row depicts only minor lobe deformation, leading to better registration accuracy.

### 3.3 Discussion

The motivation for this benchmark is to transparently assess how far rigid registration methods can address the inherently non-rigid challenges of the surgical scenario. This evaluation not only highlights the strengths and limitations of rigid approaches but also establishes a robust guideline for developing more advanced non-rigid algorithms to fill the current gaps in surgical applications.

On the deformed IRCAD dataset, generic computer-vision algorithms stay competitive when trained on small deformations. They also remain robust under various partiality conditions, but performance degrades when partiality is excessive; for example, at 5%, the target point cloud may not contain enough information to perform the registration. Thus, partiality plays a critical role in overall robustness. However, by incorporating self- and cross-attention modules with overlap/visibility scoring, the network can explicitly identify and weigh corresponding regions between the two clouds, making this combination particularly effective for handling variations in point cloud visibility. In contrast, OGMM and traditional registration methods struggle to align complete-to-partial point clouds in low-overlap scenarios.

In the DePoll registration task, the required generalization level exceeds the current algorithms’ capabilities. This challenge arises from large deformations, anatomical differences, and significant noise. In our experiments, the models trained for rigid registration on deformed data still failed under DePoll conditions. The extreme magnitude of non-rigid deformations severely impairs the models’ ability to establish correct correspondences and achieve accurate registration, demonstrating that current algorithms cannot solve this complete-to-partial task. Moreover, the pig liver’s three-lobe structure and irregular reconstruction noise differ from the human training data and increase the task’s difficulty.

Overall, our results show that when deformations are limited and the exposed intraoperative surface covers more than 10% of the organ, TREs below 10 mm can be achieved, approaching the 5 mm accuracy typically required in surgery (Doornbos et al., 2024). Moreover, when the exposed surface increases to 25%, the TRE decreases to approximately 5 mm, reaching an acceptable level for surgical practice. Conversely, in the presence of large deformations, rigid registration alone cannot achieve low TREs. However, it remains valuable as an initialization step to bring the two point clouds into closer alignment before non-rigid refinement, or as a backbone architecture that can be extended to deformation prediction.

Both our proposed solution and LiverMatch provide a solid foundation for addressing the challenge of large deformations. These architectures predict correspondences between point clouds, which can subsequently be leveraged by external algorithms (e.g., N-ICP (Amberg et al., 2007)) to perform non-rigid registration. However, their current limitation lies in the low quality of the estimated correspondences, which hinders accurate deformation prediction. For example, in our IRCAD experiments with 10% partiality, LiverMatch achieved 37% recall and 51% precision, meaning that out of 525 ground-truth correspondences, the algorithm predicts on average 380 matches, but only 194 of these are correct. In this case, it is crucial to maximize recall and precision because incorrect correspondences severely degrade the registration. To achieve this, future work should focus on improving the modules responsible for correspondence estimation, such as exploring novel feature-processing or extraction strategies. One promising direction could be to leverage semantic cues (e.g., colour information) within the point clouds to generate more discriminative and accurate matches.

An alternative strategy to improve inference performance is patient-specific training. Current state-of-the-art methods typically rely on an agnostic approach, training on large heterogeneous datasets to generalize to unseen cases. However, this paradigm may not be optimal in surgical contexts, where anatomical variability between patients might be substantial. Since preoperative CT images are routinely acquired, synthetic datasets of deformations for each patient’s organ could be generated and used to train or fine-tune the network. In this way, the model is trained and tested on the same anatomy, reducing the burden of inter-patient generalization and requiring it to adapt only to intraoperative factors such as deformation and noise.

## 4 Conclusion

In this work, we have benchmarked deep-learning and traditional approaches for point cloud registration, offering a transparent assessment of their generalizability to real-world surgical applications. The approach involves registering two input point clouds: a complete one extracted from a preoperative 3D organ model (derived from CT or MRI scans) and a partial one reconstructed from the intraoperative stereoscopic video. Our comparison covered GMM-based, correspondence-based, correspondence-free, matching-based, and liver-specific methods, aiming to identify the shared modules that lead to top performance.

Secondly, we leveraged the backbone of one of the state-of-the-art partial-to-partial registration models, and we implemented its complete-to-partial version by incorporating the estimation of overlap points only for the complete point cloud, which led to improved performance. We included our algorithm in the benchmark, demonstrating its competitive results under different partialities and deformations.

To stress the experimental setup, we intentionally applied rigid registration algorithms to scenarios in which the underlying anatomy may undergo non-rigid tissue deformations, to evaluate how well rigid approaches generalize beyond their modelling assumptions. Remarkably, despite this mismatch, deep learning–based rigid registration methods remain robust across a wide range of partiality levels when deformations are small. We attribute this resilience to combining self- and cross-attention modules with overlap scoring. However, these same methods struggle to handle large, non-rigid deformations, as the DePoll experiment shows. Addressing such cases will require developing non-rigid registration algorithms that retain the effective components of rigid models while enhancing correspondence estimation to accommodate more extreme deformations.

## Data Availability

The datasets presented in this article are not readily available because it is derived from 3D-IRCADb-01, which is licensed under the Creative Commons Attribution-Non Commercial- No- Derivatives 4.0 International (CC BY-NC-ND 4.0), a license that prohibits redistribution of modified derivative works. Requests to access the original datasets should be directed to https://www.ircad.fr/research/data-sets/liver-segmentation-3d-ircadb-01/.
